# A CLINICAL-COMMUNITY PARTNERSHIP TO ADDRESS OLDER ADULTS’ SOCIAL DETERMINANTS OF HEALTH NEEDS

**DOI:** 10.1093/geroni/igac059.2423

**Published:** 2022-12-20

**Authors:** Cory Bolkan, Raven Weaver, Erik Geissal, Breanne Wise-Swanson

**Affiliations:** Washington State University Vancouver, Vancouver, Washington, United States; Washington State University, Pullman, Washington, United States; The Vancouver Clinic, Vancouver, Washington, United States; Area Agency on Aging & Disabilities of Southwest Washington, Vancouver, Washington, United States

## Abstract

Social determinants of health (SDH) are non-medical social needs key to reducing health disparities and improving health outcomes. Adequately identifying patients’ unmet SDH needs in primary care (PC) is a critical first step in addressing them, yet many questions remain regarding feasibility and implementation of screenings and how to effectively meet patients’ needs and improve their outcomes. With formative and process evaluation analyses, we report on the development and implementation of a community-based pilot study to proactively target high-risk, low-income, older patients with SDH needs. Over a six-month planning period, leadership from a PC clinic and a community based aging services organization (CBO) collaboratively created a shared infrastructure for in-office SDH screening by clinicians with direct referral to CBO for SDH support. The research team addressed challenges of workflow and barriers to sharing/accessing electronic health records. The pilot program will cover a 2-year period (12-month enrollment; 12-month follow-up) in which patients are screened at annual visits and followed-up in the community. In the first 6 months, 286 patients were screened, from which 34 (12%) CBO referrals were made, and nine patients were receptive to receiving more information, suggesting a need to explore patient barriers and receptiveness to services/supports. We report on lessons learned, adaptations to the pilot, efforts to increase identification of eligible patients, and strategies to enhance uptake of services beyond the traditional health care setting. Investment in health and aging services partnerships is a viable pathway to reducing health care use and spending, especially for older adult populations

